# Priority-setting for achieving universal health coverage

**DOI:** 10.2471/BLT.15.155721

**Published:** 2016-02-12

**Authors:** Kalipso Chalkidou, Amanda Glassman, Robert Marten, Jeanette Vega, Yot Teerawattananon, Nattha Tritasavit, Martha Gyansa-Lutterodt, Andreas Seiter, Marie Paule Kieny, Karen Hofman, Anthony J Culyer

**Affiliations:** a NICE International, London, England.; b Center for Global Development, Washington, United States of America (USA).; c Rockefeller Foundation, New York, USA.; d FONASA, Santiago, Chile.; e HITAP, 6th Floor 6th Building, Department of Health; Ministry of Public Health, Nonthaburi 11000, Thailand.; f Ministry of Health, Accra, Ghana.; g World Bank, Washington, USA.; h World Health Organization, Geneva, Switzerland.; i School of Public Health, University of Witwatersrand, Johannesburg, South Africa.; j University of York, York, England.

## Abstract

Governments in low- and middle-income countries are legitimizing the implementation of universal health coverage (UHC), following a United Nation’s resolution on UHC in 2012 and its reinforcement in the sustainable development goals set in 2015. UHC will differ in each country depending on country contexts and needs, as well as demand and supply in health care. Therefore, fundamental issues such as objectives, users and cost–effectiveness of UHC have been raised by policy-makers and stakeholders. While priority-setting is done on a daily basis by health authorities – implicitly or explicitly – it has not been made clear how priority-setting for UHC should be conducted. We provide justification for explicit health priority-setting and guidance to countries on how to set priorities for UHC.

## Résumé

Les gouvernements des pays à revenu faible et intermédiaire sont en train de légitimer la mise en place de la couverture sanitaire universelle (CSU), suite à une résolution des Nations Unies de 2012 sur la CSU et à son entérinement dans les objectifs de développement durable fixés en 2015. La CSU variera selon les pays, en fonction de leur contexte et de leurs besoins, ainsi qu'en fonction de la demande et de l'offre de soins. Des questions fondamentales ont ainsi été soulevées par les responsables politiques et les parties prenantes, portant notamment sur les objectifs, les utilisateurs et le rapport coût-efficacité de la CSU. Si les autorités sanitaires déterminent quotidiennement des priorités, de façon implicite ou explicite, la marche à suivre pour définir les priorités en matière de CSU n'a pas été clairement établie. Nous justifions ici la nécessité de définir explicitement les priorités dans le domaine de la santé tout en donnant des orientations aux pays pour définir les priorités en matière de CSU.

## Resumen

Los gobiernos de países con ingresos bajos y medios están legitimando la implementación de una cobertura sanitaria universal (CSU) tras un acuerdo de las Naciones Unidas acerca de la cobertura sanitaria universal en 2012 y su consolidación en los objetivos de desarrollo sostenible establecidos en 2015. Cada país tendrá una cobertura sanitaria universal distinta, según el contexto y las necesidades de cada uno, así como la oferta y la demanda de atención sanitaria. Por tanto, los responsables políticos y partes interesadas han abordado los asuntos fundamentales como los objetivos, los usuarios y la rentabilidad de la cobertura sanitaria universal. A pesar de que las autoridades sanitarias han establecido prioridades diarias (de forma implícita o explícita), no se ha aclarado cómo se debería gestionar el establecimiento de prioridades para la cobertura sanitaria universal. Se ofrece una justificación para el establecimiento de prioridades sanitarias explícitas y orientación a los países en la definición de prioridades para la cobertura sanitaria universal.

## ملخص

تتولى الحكومات في الدول الأعلى دخلاً ومتوسطة الدخل سنّ تشريعات لتنفيذ التغطية الصحية الشاملة (UHC)، التزامًا منها بقرار الأمم المتحدة بشأن التغطية الصحية الشاملة الصادر في عام 2012 واستجابةً للتحفيز عليها الوارد في أهداف التنمية المستدامة التي تم وضعها في عام 2015. وستختلف التغطية الصحية الشاملة في كل دولة طبقًا لظروفها واحتياجاتها، وطبقًا لظروف العرض والطلب في مجال الرعاية الصحية. وبناءً على ذلك، طرح مقررو السياسات والجهات المعنية بعض القضايا الأساسية مثل أهداف التغطية الصحية الشاملة، والمستفيدون منها، وفاعليتها من حيث التكلفة. وبرغم التزام الجهات المسؤولة عن الناحية الصحية بتحديد الأولويات على أساس يومي، لم تتضح – سواء ضمنيًا أو صراحةً – الكيفية التي يجب من خلالها تنفيذ إجراءات تحديد الأولويات فيما يتعلق بالتغطية الصحية الشاملة. ونحن نقدم للدول المبررات اللازمة لتحديد الأولويات الصحية صراحةً، كما نقدم لها إرشادات بشأن كيفية تحديد الأولويات فيما يتعلق بالتغطية الصحية الشاملة.

## 摘要

依据联合国 2012 年就全民医疗保险 (UHC) 提出的议案以及实施 2015 年设立的可持续发展目标，中低收入国家政府批准实施全民医疗保险 (UHC)。由于各国国情和需求，以及医疗保健的供求不同，因此 UHC 也将有所区别。因此，决策者和利益相关者提出 UHC 的目标、用户和成本效益等基本问题。尽管卫生局每日间接或直接设立优先事项，但如何确立 UHC 的优先事项仍不清楚。我们就如何确立 UHC 的优先事项，向各国政府提供明显的医疗优先事项事实和指南。

## Резюме

Правительства стран с низким и средним уровнями доходов узаконивают реализацию всеобщего охвата медико-санитарной помощью в соответствии с резолюцией ООН по этому вопросу, принятой в 2012 году, и с целями устойчивого развития, определенными в 2015 году, в которых она была закреплена. Прогресс в достижении всеобщего охвата медико-санитарной помощью различается в зависимости от особенностей и потребностей страны, а также от спроса и предложения в секторе здравоохранения. Поэтому лица, определяющие политику, и заинтересованные лица задаются основополагающими вопросами о целях, потребителях и экономической эффективности всеобщего охвата медико-санитарной помощью. Хотя расстановка приоритетов в явной или неявной форме осуществляется органами здравоохранения ежедневно, до сих пор не было ясно, каким образом следует определять приоритеты для достижения всеобщего охвата медико-санитарной помощью. В данной статье обосновывается целесообразность открытого определения приоритетов в области здравоохранения и приводится руководство для стран по расстановке приоритетов для достижения всеобщего охвата медико-санитарной помощью.

## Introduction

Universal health coverage (UHC) has been defined as “the desired outcome of health system performance whereby all people who need health services receive them, without undue financial hardship.”^1^ However, scarce resources in most countries cannot ensure that everyone obtains every beneficial health service at an affordable price. Therefore, priority-setting is required to provide a comprehensive range of key services, which are well aligned with other social goals, to which all people should have access.^2^ The question then arises: how comprehensive is comprehensive? Definitions and indicators of essential health services as well as financial protection have recently been suggested to guide countries on implementing UHC.^3^^,^^4^ Policy-makers then need to make choices about what health services to provide, for whom, at what price and at what quality.

The configuration of UHC is country specific, since the demography, epidemiology, culture and history, as well as spending requirements and available resources are different for every country. Many countries set priorities using waiting lists, by compromising on quality of care, by not providing certain services to some populations or geographic areas or by charging user fees that only some can afford to pay.^5^ Ad hoc rationing processes occur implicitly in every interaction between people and health systems, but the effects of these processes on access and equity need to be considered.

Further, ad hoc or informal priority-setting can disadvantage the least well off and distort a national health system’s ability to progress towards UHC. Many of the most effective interventions that favour the poor continue to be under-used, while less cost–effective and sometimes wasteful interventions are funded.^6^ Inequity of access to health services, especially among the worse-off, provide justification to promote UHC in countries with high levels of both health and socioeconomic inequalities, which are mostly low- and middle-income countries.^7^ Meanwhile, several high-cost, new and marginally effective medications, one driver of expenditures across health care systems,^8^ have been widely adopted especially in middle-income countries.^9^^–^^11^

This paper focuses on priority-setting for UHC, first by defining priority-setting and its dimensions, followed by an exploration of explicit national priority-setting. We conclude by discussing opportunities for more concerted action to enhance UHC goals through explicit priority-setting. We provide justification for explicit health priority-setting and guidance to countries on how to set priorities for UHC.

## What is priority-setting?

### Analysis of priorities

Priority-setting in health care has been defined as:

“the task of determining the priority to be assigned to a service, a service development or an individual patient at a given point in time. Prioritisation is needed because claims (whether needs or demands) on healthcare resources are greater than the resources available.”^12^

In the context of health systems, priority-setting is about allocation of resources to innovative high-cost medicines or new vaccines and its introduction in public health programmes; prevention, or primary care; to training community workers or specialists; about deciding which population subgroups ought to receive subsidized care; even about complex policy interventions such as introducing pay-for-performance schemes for remunerating providers.

As in the case of specific drugs or surgical procedures, establishing priorities concerning human resource capacity, infrastructure investment, provider payment or premium setting for service delivery also requires systematic consideration of available evidence. And while such evidence may be more readily available in the case of pharmaceuticals, policy-makers still need to address the same two broad sets of issues when considering more complex service delivery and policy interventions. These are: first, the relative effectiveness of rival alternative interventions and, second, the value to be placed on the outcomes for each alternative. Even in the absence of technical skills or data, a structured approach setting out the costs and benefits of each option can make trade-offs explicit, highlight assumptions and gaps in evidence and reveal values underpinning decisions. 

This process can also help ensure engagement with clients and stakeholders in the process of collating, reviewing and interpreting the evidence, making implementation and impact more likely. Decision-making processes will inevitably reflect expert judgements when data for sophisticated modelling, or the local skills for analysis, are unavailable. In such circumstances, it is important to be able to interpret evidence from other settings and assess its relevance. Core principles for planning, conduct and reporting of economic evaluations have been suggested.^13^

### Explicitly setting priorities

Priority-setting is about making explicit choices about what to fund and weighing the trade-offs between the various options in the process. All health systems set priorities: these are reflected in the technologies and services paid for and in the investments made in training and infrastructure. Whether implicitly or explicitly, driven by local players or global donors, priorities become established even in settings where the institutions, data and technical expertise for doing so effectively and fairly are weak or non-existent. Thus the question is not whether to set priorities – but how to improve priority-setting processes.

When done explicitly, those who make the decisions are more likely to be known and accountable. There are clearly set out methods and processes for weighing the trade-offs between different options and for involving various stakeholders. Positive and negative lists for surgical procedures and technologies; price controls and reimbursement regulations for drugs and devices; investing preferentially in training and remunerating family doctors; all belong to a lesser or greater extent to this category of explicit priority-setting. Such explicit resource allocation mechanisms can target different types of interventions (prevention versus treatment); levels of the health system (central versus provincial government level); different geographies (e.g. urban versus rural); different services (primary versus hospital care); different population groups (e.g. women and children or the unemployed); different complexities (e.g. dialysis services or transplantation); different diseases (e.g. infectious diseases) or different technologies (e.g. vaccines or pharmaceuticals), among others. In defining explicit priority-setting, it is also helpful to discuss what explicit priority-setting is not (Box 1).

Box 1 **Explicit priority-setting is not about:**

- Cost control or cost cutting: through explicit and scientifically robust priority-setting, more resources can be released from cost-ineffective technologies towards more cost–effective ones or towards covering more people. Furthermore, priority-setting can help make the case for increasing spending on health systems by showing the value of what can be gained. Priority-setting can contribute to defining the least damaging and most explicit ways of cutting costs.
- Technocratic one-off exercises carried out by international expert consultants insulated from politics and social values: explicit priority-setting is as much about the process followed as it is about methods and data. Without a transparent, inclusive and independent process, the results of priority-setting are unlikely to be adopted. Indeed, explicit priority-setting makes difficult decisions about trade-offs easier to defend.
- Hidden denial of access to needed services: priority-setting happens even when no one dares admit it does. Explicit priority-setting offers stakeholders a chance to review and debate the decisions and perhaps reverse them. The intent of the process is to replace behind the scenes advocacy and lobbying with explicit analysis and strong governance. It replaces potentially self-seeking decisions with open, challengeable and evidence-informed ones.
- Promoting privatization or nationalization: priority-setting happens in one form or another in public, private and mixed systems. Explicit priority-setting can benefit all systems independent of their major funding source or type of provider. Priority-setting is a way for public or private health-care payers to identify what they want to buy and, potentially, where they want to buy it from, irrespective of ownership.

On the other hand, implicit methods, though by definition hard to describe, may be ad hoc, or rely on semi-explicit strategies such as peer benchmarking or oversight or devolving responsibility to the local provider through budgetary or regulatory controls. For example, without a clear benefits package, services provided rely on the clinical judgment of individual physicians. Similarly, through waiting times or ability to pay, priorities can be implemented in an implicit and hence intrinsically unaccountable way.^14^

Explicit priority-setting processes can be challenged. In an explicit process it is clear who made which decisions, the criteria used, whether the criteria used were met, what evidence was considered and whether the evidence was adequately assessed, whether appropriate values were employed, who was consulted, whether those giving advice had significant conflicts of interest and how the various trade-offs were made. More importantly, it is easier to make improvements in the process of explicit priority-setting as compared to the implicit one.

There have been major global efforts to inform global and country decisions in health, including the World Health Organization’s (WHO’s) CHOICE initiative^15^ and its essential medicines list; the World Bank’s 1993 World Development Report;^16^ and the Disease Control Priorities Project,^17^ among others (further details available from corresponding author).

These efforts have mostly focused on technical ways of defining specific interventions or technologies as priorities for all to adopt or on developing whole lists or packages of recommended cost–effective interventions. These efforts have also been coupled with global calls to action suggesting how budgets for health ought to be allocated. Sometimes such efforts were coupled with recommendations for rule-of-thumb thresholds to be used in determining cost–effectiveness usually based on a country’s gross domestic product per capita. However, if rules of thumb are too generous compared to budgets available, this can lead to implicit rationing. Some initiatives have invested in collecting and compiling regional and national data to enable better country contextualization of decisions.^15^^,^^17^ However, there has been less emphasis on local processes for collecting necessary data and for deriving and implementing decisions including health benefits packages, which comprise a set of selected essential services that is provided by partially or fully publicly subsidized funds.^18^ As a result, many technically-focused efforts at priority-setting carry the risk of imposing opportunity costs in the form of reductions in potential health.^19^^,^^20^ A recent WHO resolution^21^ supports local capacity strengthening and local determination of health priorities.

## How to set health priorities?

This section briefly covers the practical steps for priority-setting, building on a guidebook currently being developed.^18^^,^^22^ Although these steps can be applied to many areas, to maintain the focus of priority-setting for UHC, the development of a benefits package will be used as an example.

### Defining principles and scope

Authorities and stakeholders who play a role in UHC need to identify the need for setting priorities, for example determining which services to offer as part of UHC. They need to outline the principles, such as equity, efficiency, financial protection and sustainability that are driving factors or products of UHC development. Countries can decide which principles to uphold and introduce depending on their context and social values.^23^ Each UHC scheme may have different principles and priorities that may change over time depending on the health system context, yet the force behind identification and use of these principles is acceptance by all parties. The scope for setting priorities is also an important factor. Some schemes may focus only on treatment services while others may include public health programmes, health promotion and disease prevention. The scope of priority-setting may be limited to technologies or individual interventions or may extend to intersectoral interventions, such as primary care infrastructure, human resources or health information systems.

### Operationalizing the principles

After defining principles and scope, technical bodies need to define criteria that reflect the identified core principles and devise the protocols to inform who does what, when and how in the priority-setting process. A review of international experiences can be useful at the stage of developing criteria associated with selected principles. For example, equity links to accessibility for different groups that have equal needs, efficiency can be associated with value for money (incremental cost–effectiveness ratio), financial protection relates to catastrophic household expenditure and sustainability is reflected in budget impact.

In terms of protocol, priority-setting broadly involves four steps, which are: (i) identifying interventions or issues to be considered; (ii) finding evidence; (iii) making decisions and (iv) making appeals. While different stakeholders may have contrasting capacities and therefore varying contributions at each stage of the process, experiences indicate that broad stakeholder participation, especially engaging civil society and the public, is important for long-term sustainability of the process.^24^

### Selection of priority issues

As countries’ health burdens evolve and new technologies are developed, identifying appropriate topics for consideration at the right time is crucial. This also ensures transparency and trust among stakeholders that will address questions or requests for justification regarding the selection of issues for consideration. Development of explicit criteria for nomination of topics can encourage stakeholder participation in the process. The criteria should be in line with core principles of UHC and simple enough for stakeholders to understand and employ. For example, the cost of assessing particular interventions that are currently not included in the UHC benefits package could address potential issues of inequity or financial burden.^24^

### Finding the evidence

Priority-setting can be difficult if evidence is not properly considered because stakeholders have different perspectives and interests. Evidence can be considered in several ways depending on available resources and the principles identified, which can include quantitative or qualitative, global or local, clinical, social or economic and primary or secondary evidence. The groups responsible for generating the evidence should have academic integrity and limited conflicts of interest. It is also important that evidence generated is comparable across interventions. In some countries with limited capacity, the government may allow industry to provide evidence, resulting in the need for mechanisms to ensure the reliability and validity of the evidence, for example, through the establishment of methodological guidelines and independent review.^25^ In cases of limited capacity and infrastructure, there may be limitations in availability of local evidence; the attempt to generate relevant evidence can thereby help build capacity for generating local data.

### Making a decision

While making decisions is commonly perceived as the only step involved in setting priorities, the process and steps beforehand, such as the identified principles, are necessary to ensure that the decisions are legitimate, transparent and consistent. It is worth noting that evidence itself is not the deciding factor and that decision-making also requires interpretation of the evidence. Qualitative evidence can be equally important as quantitative results. Decisions that are made can be ad hoc or on a one-off basis, but setting priorities for UHC requires long-term mechanisms. Many authorities assign a group of stakeholders to take part in making decisions. Committees need to be available to listen to presentations or read reports and deliberate on the issues at hand. There should be a mechanism to appeal the decisions made and criteria should be developed to ensure that there are valid reasons for making an appeal.

### Implementing the decisions

Although the implementation of decisions is not priority-setting per se, it is important because setting priorities cannot be effective without implementation. Priority-setting needs to be linked with resource allocation in health, finance, human resources and other areas. Sometimes decisions can be implemented as pilots with plans for scaling up in the future. Evidence-informed policy decisions can help guide implementation as well as monitoring and evaluation.

### Monitoring and evaluation

Monitoring and evaluation by implementers and/or external evaluators involve assessing effective priority-setting processes, which may be different from that associated with the impact of UHC and policy implementation. It also focuses on comparing the outcomes of priority-setting and the principles that were set, determining whether the principles and criteria were achieved and identifying potential modifications or changes in the priority-setting process. However, research on monitoring and evaluation of priority-setting processes is scarce.

## Challenges and limitations

### Political economy

As setting priorities is related to allocation of public resources, it is always a political matter. Different stakeholders hold different perspectives on priority-setting, for example, health professionals may view it as a threat to clinical autonomy, industry may perceive it as a barrier to introduction of new technologies in the market and patients may think of it as a limitation on access to services. Priority-setting can be controversial and the public does not always understand the need for setting priorities, particularly due to controversies brought about by the media, even where priority-setting embodies strong ethical principles.

### Resources are essential

While it is possible to highlight features of better or worse priority-setting processes, there is no universal approach to carrying out explicit priority-setting for UHC.^26^ Every country or institution needs to find its own solution that will evolve over time. To support these context-specific processes, experiences and lessons learnt need to be shared and the value of explicit priority-setting as a necessary, but not sufficient, condition articulated. Good investments are likely to be: spending less than 0.1% of the total health care budget on deciding how to spend the remaining 99.9% more wisely; improving outcomes and access; and building the technical, evidential and institutional capacity in the process.^9^ Experiences in Chile, Ghana, Thailand and the United Kingdom of Great Britain and Northern Ireland indicate that these countries only spend a small amount on the priority-setting process.^9^

### Global organizations

By sharing experiences and practices, global organizations can help raise awareness about priority-setting. They can also support politicians and decision-makers in capacity development. In 2014, the WHO General Assembly recommended that governments establish priority-setting mechanisms, with assistance from international organizations.^21^ The establishment of country coordinating mechanisms by the Global Fund and the National Immunization Technical Advisory Group supported by the vaccine alliance GAVI, are examples. Given that more countries are graduating from aid from these global donors, it is even more important for priority-setting to be implemented by countries.^27^

### Additional factors

Priority-setting cannot solve all of the challenges and barriers associated with health resource allocation. UHC requires more than priority-setting, for example, political commitment and financial resources. Priority-setting cannot overcome weak governance, but it can support transparency and accountability and other such factors that enhance good governance. Weak regulation and implementation are also barriers to achieving priority-setting goals. Priority-setting without implementation is as futile as implementation without setting priorities. Nevertheless, many countries have been able to achieve UHC without explicit priority-setting processes in the early stages. As technologies and services multiply and health system budgets grow alongside citizens’ expectations, explicit priority-setting for UHC is becoming more important.

## Conclusion

Explicit, transparent and accountable priority-setting processes that pay attention to trade-offs when deciding on the use of scarce health-care resources are both desirable conceptually and feasible in practice. While the global community has long recognized the importance of preferentially subsidizing cost–effective health interventions and products for UHC, there is still insufficient emphasis on building national capabilities that can set priorities. Better priority-setting can constructively channel the many and growing competing demands and calls for action in health.
